# Cocktail-party listening and cognitive abilities show strong pleiotropy

**DOI:** 10.3389/fneur.2023.1071766

**Published:** 2023-03-09

**Authors:** Samuel R. Mathias, Emma E. M. Knowles, Josephine Mollon, Amanda L. Rodrigue, Mary K. Woolsey, Alyssa M. Hernandez, Amy S. Garret, Peter T. Fox, Rene L. Olvera, Juan M. Peralta, Satish Kumar, Harald H. H. Göring, Ravi Duggirala, Joanne E. Curran, John Blangero, David C. Glahn

**Affiliations:** ^1^Department of Psychiatry, Boston Children's Hospital, Boston, MA, United States; ^2^Harvard Medical School, Boston, MA, United States; ^3^Research Imaging Institute, University of Texas Health Science Center, San Antonio, TX, United States; ^4^South Texas Veterans Health Care System, San Antonio, TX, United States; ^5^Department of Human Genetics, South Texas Diabetes and Obesity Institute, University of Texas Rio Grande Valley School of Medicine, Brownsville, TX, United States

**Keywords:** cocktail-party listening, genetics, genetic correlation, cognition, hearing threshold, hidden hearing loss

## Abstract

**Introduction:**

The cocktail-party problem refers to the difficulty listeners face when trying to attend to relevant sounds that are mixed with irrelevant ones. Previous studies have shown that solving these problems relies on perceptual as well as cognitive processes. Previously, we showed that speech-reception thresholds (SRTs) on a cocktail-party listening task were influenced by genetic factors. Here, we estimated the degree to which these genetic factors overlapped with those influencing cognitive abilities.

**Methods:**

We measured SRTs and hearing thresholds (HTs) in 493 listeners, who ranged in age from 18 to 91 years old. The same individuals completed a cognitive test battery comprising 18 measures of various cognitive domains. Individuals belonged to large extended pedigrees, which allowed us to use variance component models to estimate the narrow-sense heritability of each trait, followed by phenotypic and genetic correlations between pairs of traits.

**Results:**

All traits were heritable. The phenotypic and genetic correlations between SRTs and HTs were modest, and only the phenotypic correlation was significant. By contrast, all genetic SRT–cognition correlations were strong and significantly different from 0. For some of these genetic correlations, the hypothesis of complete pleiotropy could not be rejected.

**Discussion:**

Overall, the results suggest that there was substantial genetic overlap between SRTs and a wide range of cognitive abilities, including abilities without a major auditory or verbal component. The findings highlight the important, yet sometimes overlooked, contribution of higher-order processes to solving the cocktail-party problem, raising an important caveat for future studies aiming to identify specific genetic factors that influence cocktail-party listening.

## 1. Introduction

Ordering drinks at a bar, listening to announcements in an airport terminal, and chatting in a crowded space are all real-world examples of the cocktail-party problem (1), in which listeners must segregate the acoustic mixture reaching their ears into its constituent sounds and attend to the sounds of interest (2). Among the most challenging cocktail-party

problems are those involving multiple simultaneous talkers (3). These situations are also the most critical for successful real-world hearing, as listeners report difficulties in following conversations in noisy environments more than any other kind of hearing problem [e.g., (4)]. It is therefore crucial to understand why some listeners find these situations more challenging than others.

Psychoacoustical studies provide a remarkably clear picture of which acoustic features are exploited to solve the cocktail-party problem (5, 6), and functional neuroimaging studies have made considerable progress in delineating the neural implementation of cocktail-party listening [e.g., (7)], at least in typical listeners. What is less clear is how and why listeners show such large individual differences in their cocktail-party listening abilities. Possible factors include basic sound sensitivity, peripheral auditory processing, and supramodal (i.e., cognitive) abilities. Regarding sound sensitivity, hearing impairment is obviously a major limiter of all hearing abilities, including cocktail-party listening, but while hearing aids are highly effective at improving sound sensitivity and speech intelligibility in quiet or steady-state noise in hearing-impaired listeners, their benefit in more realistic, cocktail-party-like situations falls short, suggesting that simply increasing overall audibility is not sufficient to solve the cocktail-party problem (8). Furthermore, performance on multitalker cocktail-party listening tasks differs dramatically among listeners with HTs in the normal or near-normal range [e.g., (9–12)]. These findings make it clear that cocktail-party listening and sound sensitivity are quite distinct.

Regarding peripheral auditory processing, a single mechanism has garnered particular interest in recent years. Non-human animal studies have shown that cochlear synaptopathy, or loss of the connections between hair cells and auditory-nerve fibers, may degrade the temporal representations of suprathreshold sounds in the absence of elevated HTs (13). Cochlear synaptopathy can be induced by moderate amounts of noise exposure [e.g., (14)] and occurs naturally *via* normal aging [e.g., (15)]. Several non-invasive electrophysiological correlates of cochlear synaptopathy have been proposed, which can be applied to living humans (16, 17). However, cochlear synaptopathy as an explanation of poor cocktail-party listening in humans remains controversial because the extent to which synaptopathy correlates with multitalker listening tasks or self-reported real-world hearing problems is unclear [e.g., (18)].

Prior studies make it abundantly clear that listeners' cocktail-party listening abilities correlate with their cognitive abilities. Studies reporting such correlations have employed a variety of experimental designs, ranging from comprehensive psychophysical and cognitive assessments in small samples [e.g., (19)] to cursory assessments in huge samples [e.g., (20)]. In a metaanalysis of 25 individual studies, Dryden et al. (21) estimated an overall moderate correlation between speech-in-noise performance and cognitive abilities. Their analysis collapsed across various speech-in-noise tasks, cognitive measures, and listeners with and without hearing impairment. The authors concluded that there were not clear differences in correlations as a function of the target stimulus or masker type, but they did conclude that some cognitive domains were more strongly correlated with speech-in-noise performance than others: the cognitive domains considered, in order of strongest to weakest correlation, were processing speed, inhibitory control, working memory, episodic memory, and crystallized intelligence. These conclusions should be interpreted somewhat cautiously, as Dryden et al. noted, because of the considerable heterogeneity in study designs [similarly, see (22)]. Nevertheless, it is interesting to note that these correlations did not seem to be stronger for cognitive tasks with a prominent auditory or verbal component, suggesting that these relationships were not due to common method variance (23).

Previously, we explored whether genetic factors influenced cocktail-party listening (24). We measured speech-reception thresholds (SRTs) in a cocktail-party listening task where listeners reported target sentences mixed with time-reversed masker sentences from different talkers. Listeners were recruited from large pedigrees as part of the Imaging Genomics of the Aging Brain (IGAB) study. Quantitative genetic analyses suggested that just over half of the variance of SRTs was due to additive genetic factors. This estimate of heritability did not appear to be influenced by environmental factors that were shared among relatives (e.g., current household), and was robust to the inclusion and exclusion of hearing-impaired listeners. Furthermore, the genetic correlation between SRTs and HTs, or the correlation between their latent additive genetic influences, was not significantly different from 0, although it was significantly different from 1. This result suggested that the genetic factors influencing cocktail-party listening were largely distinct from those influencing sound sensitivity, which was consistent with the idea that normal sound sensitivity is not sufficient to solve the cocktail-party problem, as mentioned earlier. Overall, the findings suggested that future studies could identify specific genetic variants that influence cocktail-party listening—and by extension, real-world hearing problems—in listeners without clinical hearing impairment.

It remains to be established whether the genetic factors influencing cocktail-party listening overlap with those influencing cognitive abilities. In the present study, we explored this open question by estimating the phenotypic and genetic correlations between SRTs, HTs, and various cognitive abilities in listeners from the IGAB study. This sample was randomly ascertained with respect to hearing, meaning that some of them had hearing loss. The sample also represented a cross-section of the adult lifespan, including both young and old adults. Our primary aim was to estimate correlations between SRTs, HTs, and cognitive abilities. Although such correlations have been estimated before [cf. (21)], all previous studies estimated phenotypic correlations only. Other novel features of the present study were that we measured a wide range of cognitive abilities, rather than focusing on just one or a few specific tasks or abilities [e.g., working memory; (25)], and the sample size was large compared to other studies that measured many cognitive abilities in the same listeners [e.g., (19)].

## 2. Materials and methods

### 2.1. Listeners

The IGAB study recruited 493 listeners, 304 of whom were genetically female. Listeners ranged from 17 to 91 years old, with a median age of 47.8 years, and belonged to 54 pedigrees of varying size. The largest pedigree had 91 members. Reported familial relationships were verified based on autosomal markers.

Listeners were not recruited or excluded based on any criteria except that they must have participated in at least one prior genetic study. These studies were the San Antonio Family Heart Study [SAFHS; (26)]; the San Antonio Family Gallbladder Study [SAFGS; (27)]; and the Genetics of Brain Structure and Function Study [GOBS; (28)]. SAFHS occurred across three recruitment phases between 1992 and 2007. To be eligible for SAFHS, an individual had to be Mexican American, aged 40–60 years, have a spouse willing to participate, and have at least six adult (>16 years old) offspring and/or siblings. SAFHS also recruited the spouses of these participants (if they were Mexican American), their first-, second, and third-degree adult relatives, and Mexican American spouses of those relatives. SAFGS was conducted between 1998 and 2001 and recruited additional Mexican American families in a similar way, except that the initial proband always had type-2 diabetes. Since this disorder has a lifetime prevalence approaching 30% in this population, the recruitment strategy employed in SAFGS represented effectively random sampling for other diseases, behaviors, and abilities. GOBS was conducted between 2006 and 2016 and re-recruited SAFHS and SAFGS individuals, as well as their previously unrecruited adult offspring. Thus, all listeners were sampled from the same community.

All listeners provided written informed consent on forms approved by the institutional review board at the data-collection site, University of Texas Health Science Center at San Antonio, as well as review boards at the University of Texas Rio Grande Valley and Boston Children's Hospital.

### 2.2. Overview of the assessments

We attempted to conduct the auditory and cognitive assessments described in the following sections on all listeners in the IGAB study. Usually, a listener completed these assessments during a single laboratory visit, although occasionally a listener was unable to complete one or more of them, for various reasons. During the same visit, listeners completed a brief structured interview to determine their medical histories, the mini-mental state examination [MMSE; (29)], and the clinical dementia rating (CDR) staging instrument (30). Listeners completed other assessments to collect demographic information, physical variables, and biological samples, but these were not relevant to the present goals and are not described here.

Most listeners spoke English as their first language and their assessments were conducted in English. However, a small proportion of listeners spoke Spanish as their first language, and these individuals completed Spanish translations or versions of each assessment if such a translation/version was available. Spanish translations/versions were available for most cognitive assessments, but notably not the cocktail-party listening task. We therefore only analyze data from English-speaking listeners here (see the next section).

Auditory and cognitive assessments were performed under the supervision of a member of the research team in a quiet testing room using a laptop with an integrated digital-to-analog converter and a touchscreen display. The cocktail-party listening task and hearing test were conducted with connected headphones (Sennheiser HD 25 Pro), while the cognitive tests used the laptop's integrated loudspeakers. Listeners made their responses using the keyboard, the touchscreen, or orally, depending on the assessment.

### 2.3. Exclusions

During their medical interviews, one listener reported multiple sclerosis, two reported Parkinson's disease, two reported Alzheimer's disease, one reported non-Alzheimer's dementia, 15 reported strokes, and three reported another neurological disorder or brain trauma. Several listeners were suspected to have at least mild cognitive impairment based on the neurological assessments: 12 listeners scored below 24 on the MMSE, and three listeners had CDR global scores and/or sum of boxes scores above 1 and/or 4.5, respectively. It became apparent during their assessments that eight listeners were illiterate. Six listeners were Spanish speakers. While none of the above features were exclusion criteria for the IGAB study *per se*, we have excluded these listeners from the present study (40 exclusions in total).

### 2.4. Cocktail-party listening task

For several reasons outlined in our previous article (24), we opted to develop a novel cocktail-party listening task using synthetic speech and time-reversed maskers. Briefly, the task was time-efficient, as listeners made multiple responses to a single brief sentence per trial [cf. (31)], and performance could not be improved by paying attention to the syntactic structure or semantic content of the sentences [cf. (32)]. Synthetic speech using realistic voice models (33) allowed the construction of a very large corpus with coarticulation across words, and reversed maskers prevented some listeners from becoming confused about the task demands.

The task was similar to the every-other-word paradigm devised by Kidd et al. (34). On each trial, the listener heard a target sentence starting with the name “Jane” followed by four variable words: a verb, a number, an adjective, and a noun. There were eight possible variable words per position (verbs: “bought,” “found,” “gave,” “heard,” “held,” “kicked,” “saw,” “threw;” numbers: “two” to “ten” excluding “seven;” adjectives: “big,” “blue,” “cold,” “hot,” “black,” “old,” “red,” “small;” nouns: “bags,” “cards,” “gloves,” “hats,” “pens,” “shoes,” “socks,” “toys”). Listeners reported the variable words per target sentence *via* a graphical user interface on the touchscreen display, with one button per word.

Target sentences were presented at an average sound pressure level (SPL) of 60 dB and mixed with two random masker sentences constructed from the same corpus but with a different name (“Pat” and “Sue”) and with the constraint that no word could occur more than once on a given trial. Masker SPLs were manipulated to achieve a desired signal-to-noise ratio (SNR) with the targets. Maskers were time-reversed and aligned to have simultaneous onsets with the targets. All sounds were presented diotically.

On the first trial of the task, the SNR was 40 dB (i.e., maskers were 20 dB SPL). On following trials, SNRs were decreased and increased by 2 dB for every correct and incorrect selection, respectively, on the immediately preceding trial. For example, if a listener selected three variable words correctly (i.e., made one error) on the first trial, the SNR on the second trial was 40 – 2 – 2 – 2 + 2 = 36 dB. It is straightforward to show that this procedure converges asymptotically on the SNR value that yields a 50% chance of a correct response, assuming a constant psychometric function (35). The task was always terminated after 30 trials. SRTs were estimated by taking the mean of all SNR values excluding the SNR on the first trial, which was always 40 dB and therefore uninformative, and including the theoretical 31st trial, whose SNR could be calculated based on listeners' responses to the 30th trial.

### 2.5 Hearing test

As described in our previous article (24), the hearing test measured HTs for 0.5-, 1-, 2-, 4-, 8-, and 12.5-KHz pure tones in both ears. Each trial in the hearing test comprised a 2-s interval which equiprobably contained or did not contain a monaural 1-s pure tone whose amplitude was modulated at 100% depth using a 2-Hz full-wave rectified sinusoid. On each trial, listeners pressed the space bar if they heard a tone during the interval. Trials were organized into separate blocks for each frequency and ear. The lowest frequency tested was 0.5 KHz because previous work suggests that HTs measured inside and outside of a sound-attenuated chamber are largely equivalent at or above this frequency, whereas lower-frequency HTs may be unreliable (36). Within a block, the first tone had a fixed level of 60 dB hearing level (HL) and the levels of subsequent tones were manipulated using a single interval adjustment matrix (37) with an adjustment factor of 10 dB up to the second reversal and 4 dB afterward. Blocks were terminated after six reversals. HTs were defined as the quietest sound heard per frequency and ear. Better-ear average (BEA) HTs were calculated using all frequencies except 12.5 KHz.

### 2.6. Cognitive tests

Cognitive assessments were administered using the latest version of our in-house computerized cognitive battery, Charlie, which we have used in prior studies [e.g., (38, 39)], and is the successor of the South Texas Assessment of Neurocognition (STAN), which was used in the GOBS study [e.g., (40)]. Charlie contains many of the same tests as STAN but was updated to run using modern hardware (e.g., touchscreen computers). Individual tests and their associated dependent variables are described below. Tests were completed in the order they are described.

#### 2.6.1. Orientation

The first test in the battery was a simple measure of visual search speed. On each trial, a red square appeared in a random position on the touchscreen and listeners touched the square as quickly as possible. There were 15 such trials in total. This test was originally developed to introduce the listener to the touchscreen device and ensure that they could operate it correctly (hence the name “orientation”), but we found that it yielded meaningful cognitive data in a previous study (39). The test yielded a single dependent variable, namely the log-transformed time taken to complete all trials.

#### 2.6.2. Trail-making test (TMT) part A

This test was a computerized analog of part A of the classic trail-making test (41), which measures visual search and processing speed. During the test, numbers 1 to 26 appeared inside circles that were randomly positioned on the touchscreen. Listeners touched the circles, one by one, in ascending numerical order, as quickly as possible. After touching an appropriate circle, a line appeared that connected the current circle to the previous circle, forming a trail between them. Upon touching an incorrect circle, listeners heard a brief feedback sound instead. The tested ended after the final circle was touched. The dependent variable was the log-transformed completion time.

#### 2.6.3. TMT letter

This test was identical to the TMT part A, except that the circles contained letters of the alphabet instead of numbers, and listeners touched them in ascending alphabetical order. It was intended to serve as an intermediate condition between parts A and B of the classic trail-making test, since poor performance on part B could be caused by poor literacy. Again, the dependent variable was the log-transformed completion time.

#### 2.6.4. TMT part B

This test was a computerized analog of part B of the classic trail-making test, which measures set shifting and executive functioning. Twenty-six circles, each containing a number or letter, appeared in random positions on the screen. Listeners touched them in alternating ascending numerical and ascending alphabetical order (1, “a,” 2, “b” …) as quickly as possible. The dependent variable was the log-transformed completion time.

#### 2.6.5. Matrix reasoning

This test used the same stimuli as the progressive matrix-reasoning test that appears in the Wechsler adult intelligence scale [WAIS; (42)], which measures non-verbal abstract reasoning. On each trial, listeners saw a visual puzzle or matrix with a piece missing, and touched the missing piece from four alternatives presented below it. The dependent variable was the total number of correct responses.

#### 2.6.6. Visuospatial memory

This test measured visuospatial short-term memory capacity using a change-localization test, similar to the one used by Johnson et al. (43). On each trial, four items with random shapes, positions, and colors appeared on the touchscreen for a brief period, then disappeared for a longer period. After the second period, three of the items reappeared, and a fourth item with a novel shape and color appeared in the position previously occupied by the missing item. Listeners touched the new item. The dependent variable was the total number of correct responses.

#### 2.6.7. Emotion recognition

This test was identical to the ER-40, which is widely used in psychiatry research to index the ability to judge emotions in facial expressions (44). On each trial, listeners saw a color photograph of a static face expressing a happiness, sadness, anger, fear, or no emotion. Listeners touched the word describing the corresponding emotion from the five alternatives. The dependent variable was the total number of correct responses.

#### 2.6.8. California verbal learning test

This test was a modified and abridged version of the adult CVLT, second edition (45), which measures episodic verbal learning and memory. On each trial, listeners heard 16 words spoken aloud and then repeated out loud as many of them as possible. Oral responses were recorded by the administrator. There were five trials, and the same 16 words were heard in the same order each time. The dependent variable was the total number of correct responses summed over trials.

#### 2.6.9. Forward span

This classic measure of verbal short-term memory capacity is found in many standardized cognitive batteries, such as the WAIS. Listeners heard sequences of digits and repeated them out loud. Oral responses were recorded by the administrator. The dependent variable was the improved mean span metric proposed by Woods et al. (46).

#### 2.6.10. Backward span

This is a more challenging variant of forward span in which listeners repeated sequences of digits in reverse order. Oral responses were recorded by the administrator. The dependent variable was the improved mean span metric.

#### 2.6.11. Letter–number sequencing

This is the classic measure of verbal working memory capacity—as opposed to short-term memory capacity, since it requires the ability to manipulate as well as recall remembered items —found in many cognitive batteries, including as the WAIS. Listeners heard sequences of letters and digits, and repeated them back in alternating ascending numerical and alphabetical order. Oral responses were recorded by the administrator. The dependent variable was the improved mean span metric.

#### 2.6.12. Wechsler test of adult reading

This is a widely used test of reading ability (47). Listeners attempted to correctly pronounce words from a list of 50 words of increasing difficulty. Oral responses were recorded by the administrator. The dependent variable was the total number of correct responses.

#### 2.6.13. Controlled oral word association test letter

This is the traditional “fas” variant of the COWAT, which measures verbal fluency (48). Over three trials, listeners said as many unique real words beginning with a specific letter as possible, discounting proper nouns, in 1 min. The letters were “f,” “a,” and “s” on the first, second, and third trials, respectively. Oral responses were recorded by the administrator. The dependent variable is the total number of valid responses.

#### 2.6.14. COWAT animal

This is another variant of the COWAT, which measures semantic verbal fluency (49). Listeners named as many unique animals as possible in 1 min. Oral responses were recorded by the administrator. The dependent variable is the number of valid responses.

#### 2.6.15. Digit symbol

This is a two-alternative forced-choice computerized variant of the digit–symbol substitution test (38), which measures processing speed. Listeners were presented with a key of symbols and digits at the top of the screen, which persisted across all trials. On each trial, they saw a new random digit and random symbol, and judged whether they made a correct pair according to the key. The dependent variable is the number of correct responses made within two 90-s blocks, multiplied by overall accuracy; the multiplicative term served to penalize individuals who responded quickly but with poor accuracy.

#### 2.6.16. Facial memory

This test measures facial recognition memory. During a learning phase, listeners saw 20 monochrome photographs of strangers' faces, presented sequentially. During a recognition phase, listeners were presented with faces, one per trial, that were equiprobably one of those from the learning phase or entirely novel. On each trial, listeners made an old/new judgement. The dependent variable is the number of correct responses.

#### 2.6.17. Continuous performance test

This is the identical-pairs version of the widely used continuous performance test, which measures sustained attention (50). On each trial, listeners see a row of three random symbols for a brief period and respond when all three symbols match those from the immediately preceding trial. The dependent variable is the number of hits, or matches correctly reported.

#### 2.6.18. Logical memory

This was identical to the logical memory test from the Wechsler memory scale (51), which measures verbal episodic memory. This test contained three parts. In the first part, listeners immediately recalled details of two short passages. In the second part, listeners recalled the passages after a delay. In the third part, listeners answered yes or no questions regarding the passages. The dependent variable was the total raw score.

### 2.7. Quantitative genetic analysis

#### 2.7.1. Univariate models

A univariate quantitative genetic model attempts to explain the phenotypic (or observed) variance of a single focal trait in terms of ensemble genetic and environmental factors. Under the standard assumptions of quantitative genetics (52), the focal trait vector, denoted by ***y***, follows a multivariate normal distribution, ***y*** ~ *N*(**μ**, **Ω**). The mean of this distribution, denoted by **μ**, is given by **μ** = ***X**β*, where ***X*** is a design matrix of fixed-effect nuisance covariates, such as age and sex, and **β** is a vector of their corresponding regression coefficients. The covariance matrix, denoted by **Ω**, is given by **Ω** = 2**Φ**$\sigma_{\text{G}}^{2}$ + $\text{I}\sigma_{\text{E}}^{2}$, where **Φ** is the matrix of kinship coefficients between listeners (determined by their pedigrees), $\sigma_{\text{G}}^{2}$ is the additive genetic variance (a free parameter), ***I*** is an identity matrix, and $\sigma_{\text{E}}^{2}$ is the environmental or residual variance (another free parameter).

Narrow-sense heritability (53) is given by *h*^2^ = $\sigma_{\text{G}}^{2}$ / ($\sigma_{\text{G}}^{2}$ + $\sigma_{\text{E}}^{2}$) and can be thought of as an effect size for the genetic effect, as it represents the proportion of phenotypic variance explained by additive genetic factors. For example, if *h*^2^ = 1, the trait would be completely determined by such factors; if h *h*^2^ = 0.5, half the trait's phenotypic variance would be determined by such factors. Because we often wish to test the statistical significance of *h*^2^, it can be convenient to reparameterize the equation for the covariance matrix as **Ω** = [2**Φ***h*^2^ + ***I*** (1 – *h*^2^)]σ^2^, so that *h*^2^ and the phenotypic standard deviation, denoted by σ, are free parameters. This allows us to construct a null model where *h*^2^ = 0. The null and alternative models are both fitted to the data *via* maximum likelihood estimation, and a likelihood ratio test (LRT) is constructed to obtain a *p*-value for the test of heritability.

We fitted univariate quantitative genetic models to SRTs, HTs, and the 18 individual cognitive measures (i.e., 20 models in total). Fitting was done using the SOLAR software package (54). The purpose of these analyses was to check if all traits were heritable, as we expected based on previous studies. Before model fitting, traits were rank-based inverse-normal transformed to ensure that they were normally distributed and reduce the influence of outliers. All models contained an intercept, age, age^2^, sex, an age × sex interaction, and an age^2^ × sex interaction as fixed-effect covariates. All of these fixed effects were included in every model, including bivariate and trivariate models (described below), regardless of their statistical significance.

#### 2.7.2. Bivariate models

A bivariate quantitative genetic model is an extension of a univariate model that considers two traits simultaneously. The equations are available elsewhere [e.g., (52)]. Crucially, bivariate models provide not only heritability estimates for two traits, but also estimates of their phenotypic, genetic, and environmental correlations. The phenotypic correlation, denoted by ρ_P_, is the correlation between the phenotypes (i.e., observed values)—it is exactly like the more commonly understood Pearson's product–moment coefficient and its values can be interpreted the same way; for example, ρ_P_ = 0 represents independence and ρ_P_ = ±1 represents complete correlation. The genetic correlation, denoted by ρ_G_, describes the correlation between the traits' latent additive genetic factors. Again, ρ_G_ = 0 represents independence (of the underlying genetic factors) and ρ_G_ = ±1 represents complete correlation (between the genetic factors, also called complete pleiotropy). Note that ρ_P_ and ρ_G_ are guaranteed to converge only when both traits are perfectly heritable; therefore, ρ_G_ can be exactly ±1, implying complete pleiotropy, even if ρ_P_ is not, due to non-genetic factors (e.g., measurement error) influencing the traits. Finally, the environmental correlation, denoted by ρ_E_, describes the correlation between the traits' non-genetic components. Since measurement error is a major non-genetic component, environmental correlations are the most difficult to interpret (and often the least interesting) of the three correlation types.

Under the default parameterization, ρ_G_ and ρ_E_ are free parameters, allowing null models where ρ_G_ = 0 or ρ_E_ = 0 to be fitted and LRTs to determine whether traits are significantly genetically or environmentally correlated. Another possibility is to test whether traits show incomplete pleiotropy, using a null model where ρ_G_ = ±1. While ρ_P_ can be estimated deterministically, the model also can be reparameterized so that ρ_P_ is a free parameter, which allows an LRT of phenotypic correlation.

We fitted bivariate models in which one trait was always SRTs, and the other was either HTs or an individual cognitive measure (i.e., 19 models in total). Per model, we performed LRTs to test whether ρ_P_ differed from 0, ρ_G_ differed from 0, ρ_G_ differed from ±1, where the sign matched that of the ρ_G_ estimate, and ρ_E_ differed from 0.

Bivariate models can handle incomplete data; that is, when one individual has a value for one trait but not the other, allowing maximal use of all available data.

#### 2.7. 3. Endophenotype ranking

The endophenotype ranking value (ERV) is a helpful metric for ranking trait pairs (40). It is defined deterministically as ERV = |√$h_{1}^{2}\surd$$h_{1}^{2}$ρ_G_|, where $h_{1}^{2}$ and $h_{2}^{2}$ are heritabilities of two traits. This quantity represents the phenotypic covariance of the traits explained by the same genetic factors, and balances the strengths of the genetic signals and the strength of their genetic relationship. It is sometimes called bivariate heritability (55). We estimated ERVs for all SRTs and HTs, as well as all SRT–cognition trait pairs (19 ERVs in total).

#### 2.7.4. Correction for multiple comparisons

All *p*-values were corrected for multiple comparisons by applying a single-step false-discovery rate (FDR) adjustment at the 0.05 level (56).

## 3. Results

### 3.1. Heritabilities

Table 1 shows narrow-sense heritability estimates for all traits. SRT and HT heritability estimates (*h*^2^ = 0.553 and *h*^2^ =0.337, respectively) were extremely similar to those we reported previously in a slightly smaller sample of the same listeners (24). Cognitive measures had a range of heritabilities, with orientation being the weakest (*h*^2^ = 0.239) and WTAR being the strongest (*h*^2^ = 0.770). This pattern of heritability estimates for cognitive traits was consistent with the pattern we reported in the GOBS study, which was conducted about a decade ago and involved the same individuals and their close relatives (40, 57). All heritabilities were significantly >0 at the FDR-corrected level (5.12 ≤ χ^2^ ≤ 48.5; 1.62 × 10^−12^ ≤ *p* ≤ 0.0118; 1.01 × 10^−11^ ≤ *p*_FDR_ ≤ 0.0174).

**Table 1:** Results from the univariate and bivariate quantitative genetic analyses.

**Trait**	***N***	**h2**	**ρP**	**ρG**	**ρE**	**ERV**
SRTs	400	**0.553 (0.135)**				
COWAT letter	442	**0.534 (0.117)**	**−0.436 (0.0424)**	**−0.980 (0.153)**	0.0977 (0.181)	0.532
Digit symbol	438	**0.598 (0.123)**	**−0.409 (0.0452)**	**−0.864 (0.100)**	0.323 (0.277)	0.496
TMT part B	439	**0.460 (0.125)**	**0.442 (0.0424)**	**0.906 (0.143)**	0.0109 (0.179)	0.457
Logical memory	342	**0.689 (0.149)**	**−0.429 (0.0480)**	**−0.698 (0.121)** ^*^	0.0683 (0.303)	0.431
TMT letter	441	**0.357 (0.104)**	**0.416 (0.0436)**	**0.963 (0.146)**	0.0408 (0.144)	0.428
LNS	435	**0.329 (0.110)**	**−0.458 (0.0410)**	**−1 (n/a)**	−0.0524 (0.147)	0.426
CVLT	405	**0.676 (0.142)**	**−0.435 (0.0434)**	**−0.696 (0.143)** ^*^	−0.0462 (0.235)	0.426
Backward span	416	**0.514 (0.119)**	**−0.438 (0.0439)**	**−0.745 (0.128)**	−0.0821 (0.191)	0.397
WTAR	406	**0.770 (0.109)**	**−0.508 (0.0400)**	**−0.570 (0.121)** ^*^	−0.448 (0.193)	0.372
Orientation	442	**0.239 (0.120)**	**0.240 (0.0481)**	**1 (n/a)**	−0.165 (0.143)	0.363
CPT	356	**0.416 (0.150)**	**−0.242 (0.0523)**	**−0.729 (0.172)** ^*^	0.244 (0.205)	0.349
TMT part A	441	**0.267 (0.106)**	**0.373 (0.0465)**	**0.900 (0.149)**	0.0449 (0.144)	0.345
Matrix reasoning	432	**0.414 (0.125)**	**−0.427 (0.0427)**	**−0.708 (0.159)** ^*^	−0.186 (0.156)	0.339
COWAT animal	442	**0.692 (0.105)**	**−0.330 (0.0472)**	**−0.538 (0.149)** ^*^	−0.0231 (0.207)	0.333
Forward span	432	**0.400 (0.130)**	**−0.420 (0.0433)**	**−0.696 (0.196)** ^*^	−0.234 (0.141)	0.327
Facial memory	442	**0.406 (0.127)**	**−0.421 (0.0433)**	**−0.648 (0.142)** ^*^	−0.202 (0.164)	0.307
Emotion recognition	409	**0.394 (0.122)**	**−0.340 (0.0462)**	**−0.634 (0.152)** ^*^	−0.0691 (0.166)	0.296
Visuospatial memory	433	**0.257 (0.122)**	**−0.389 (0.0438)**	**−0.698 (0.219)** ^*^	−0.230 (0.136)	0.263
HTs	405	**0.337 (0.131)**	**0.311 (0.0472)**	0.362 (0.210)^*^	0.284 (0.151)	0.156

h^2^, narrow-sense heritability; ρ_*P*_, phenotypic correlation; ρ_*G*_, genetic correlation; ρ_*E*_, environmental correlation; ERV, endophenotype ranking value; SRT, speech reception threshold; COWAT, controlled oral word association test; TMT, trail-making test; LNS, letter–number sequencing; WTAR, Wechsler test of adult reading; CPT, continuous performance test; CVLT, California verbal learning test; HT, hearing threshold. In the third, fourth, and sixth columns from the left (heritabilities, phenotypic correlations, and environmental correlations, respectively), the leftmost value in each cell is the parameter estimate, the parenthetical is the standard error of that estimate, and a bold value indicates that an estimate was significantly different from 0 at the FDR-corrected level. The same is true for the fifth column from the left (genetic correlations), except that an asterisk also indicates that an estimate was significantly different from ±1 (whichever is closer to the parameter estimate). Note that sometimes the ρ_*G*_ estimate was exactly ±1: in these cases, the estimate converged to a parameter boundary and standard errors could not be computed (hence “n/a”), though statistical tests could still be performed.

### 3.2. Phenotypic correlations

The phenotypic correlation between SRTs and HTs was positive, indicating that larger (worse) SRTs were associated with larger (worse) HTs, and significantly different from 0 at the FDR-corrected level [ρ_P_ = 0.311; $\chi_{\left(1,\text{N}=405\right)}^{2}$ = 35.8; *p* = 2.14 × 10^−9^; *p*_FDR_ = 1.06 × 10^−8^]. This is consistent with our previous study (24).

Phenotypic SRT–cognition correlations ranged from weak (SRT–orientation ρ_P_ = 0.240) to strong (SRT–WTAR ρ_P_ = −0.508), but most of them were stronger than the SRT–HT correlation (see Table 1). All SRT–cognition correlations were significantly different from 0 at the FDR-corrected level (19.8 ≤ χ^2^ ≤ 94.2; 2.84 × 10^−22^ ≤ *p* ≤ 8.42 × 10^−6^; 2.67 × 10^20^ ≤ *p*_FDR_ ≤ 2.40 × 10^−5^). SRT–cognition correlations were negative for all cognitive measures that were based on accuracy, where a lower score reflected poorer performance, and positive for all time-based measures, where a larger score indicated worse performance.

### 3.3. Genetic correlations

The genetic correlation between SRTs and HTs was positive, but not significantly different from 0 at the FDR-corrected level [ρ_G_ = 0.362; $\chi_{\left(1,\text{N}=405\right)}^{2}$ = 2.36; *p* = 0.125; *p*_FDR_ = 0.161]. However, it was significantly different from 1 [$\chi_{\left(1,\text{N}=405\right)}^{2}$ = 7.74; *p* = 0.161; *p*_FDR_ = 0.00445]. In other words, the hypothesis of no pleiotropy could not be rejected, but the hypothesis of complete pleiotropy could, suggesting that the genetic influences on SRTs and HTs were at least partially distinct. This result is consistent with our previous study (24).

All genetic SRT–cognition correlations were strong and significantly different from 0 at the FDR-corrected level (0.538 ≤ |ρ_P_| ≤ 1; 7.19 ≤ χ^2^ ≤ 28.7; 8.60 × 10^−8^ ≤ *p* ≤ 0.00734; 4.04 × 10^−7^ ≤ *p*_FDR_ ≤ 0.0113). SRT–LNS and SRT–orientation correlations were estimated to be exactly ±1 (a parameter boundary). Genetic correlations were always in the same direction as their corresponding phenotypic correlations, but were always stronger. For some cognitive measures, the correlation was not significantly different from ±1 at the FDR-corrected level.

### 3.4. Environmental correlations

None of the environmental correlations were significantly different from 0.

### 3.5. ERV ranking

Traits are presented in descending order of their ERV in Table 1. COWAT letter, digit symbol, TMT part B and logical memory had the highest ERVs, whereas visuospatial memory, emotion recognition, facial memory, and forward span had the lowest, although the range was rather narrow (see Table 1). All cognitive measures outranked HTs in terms of ERVs.

## 4. Discussion

In a previous study, we found that SRTs were heritable (24). That study as well as previous studies also found that HTs were heritable [e.g., (58, 59)]. Although it was not our goal to replicate such discoveries here, the results of the present study were entirely consistent with these previous findings. It is already well established that cognitive abilities are heritable, and the pattern of heritability estimates in the present study were similar to those in a previous family study we conducted a decade ago (40, 57). In the present study, as in other quantitative genetic studies, the goal was not to identify associations between specific genetic variants and these traits. Therefore, the results do not tell us which genes are involved in cocktail-party listening, sound sensitivity, or cognitive abilities. However, significant heritability estimates do suggest that such genes exist and are potentially discoverable *via* techniques such as linkage or association analysis, which we have applied previously to cognitive abilities [e.g., (57, 60)]. We intend to conduct such analyses on hearing traits in future studies.

As we found in our previous study, both phenotypic and genetic correlations between SRTs and HTs were modest (24). Only the phenotypic correlation was significantly different from 0, though the genetic correlation was significantly different from 1. Thus, while SRTs and HTs were at least phenotypically correlated, there was at most a modest overlap in their genetic factors. These results lend further support to the idea discussed earlier, namely that in groups of listeners with typical HTs, sound sensitivity does not play a critical role in cocktail-party listening. Our findings also extend this idea by suggesting that the genetic factors influencing cocktail-party listening are mostly different from those influencing sound sensitivity in such samples. This line of reasoning may lead to two further speculations. The first is that future genetic studies could seek to identify specific genetic factors for cocktail-party listening abilities in samples of people without (or at least, not ascertained for) clinical hearing impairment. The second is that it complicates the interpretation of studies that do not explicitly disentangle cocktail-party listening and sound sensitivity. For instance, a genome-wide association study conducted in the UK Biobank identified several risk loci for self-reported hearing problems (61). However, because this study did not measure HTs, people in the affected group were probably a mix of listeners with clinical hearing impairment and listeners who experienced hearing problems yet had normal HTs [e.g., (62)]. The authors compensated for this limitation by performing an additional association analysis of hearing-aid use. As expected, this second analysis yielded some but not all the same loci as the first. Importantly, the results of this study were somewhat different to those of other genome-wide association studies in which listeners' medical records were available and therefore included confirmed cases of clinical hearing impairment, or studies where HTs were available [e.g., (63)]. Thus, there is a clear need for objective measures of both SRTs and HTs in future genetic studies.

The main finding of the present study was that SRTs were strongly genetically correlated with all cognitive abilities. Some of these correlations could not be distinguished from ±1 statistically. Others were estimated to be exactly ±1, which can happen under quantitative genetic models because the optimization procedure hits a parameter boundary; these estimates would likely converge away from the boundary given more data. From these results, we conclude that there is extremely strong pleiotropy between SRTs and cognitive abilities, perhaps as much pleiotropy as between pairs of cognitive abilities. All genetic SRT–cognition correlations were stronger than the genetic correlation between SRTs and HTs—we found this result very surprising, as we expected the opposite to be true a priori.

When we ranked cognitive measures by their ERVs, or covariance with SRTs explained by shared genetic factors, a measure of verbal fluency (COWAT letter) came out on top, followed by a measure of processing speed (digit symbol), a measure of set shifting and processing speed (TMT part B), and a measure of verbal episodic memory (logical memory). It is interesting that at least two of the four measures involved processing speed—digit symbol and the TMT are classic processing-speed measures, and one could argue that the COWAT relies on processing speed as well, as it requires making verbal responses as quickly as possible. This is consistent with the metanalysis by Dryden et al. (21). Processing speed is more susceptible to age-related decline than any other cognitive domain (64), raising the possibility that the commonly observed age-related increases in SRTs (8, 65) could be tied to older listeners' declining processing speed. Two of the four tests (COWAT and logical memory) involved recalling verbal information from long-term memory; it is not immediately clear why such tasks would outrank those involving verbal working memory. The lowest-ranked measures (visuospatial memory, emotion recognition, facial memory, and TMT part A) were all primarily visual in nature, although the difference between the smallest and largest ERV was not enormous.

The role of cognitive abilities in cocktail-party listening has been explored in previous studies. Some studies of this kind have focused on a single cognitive domain, such as verbal working memory [e.g., (25)], and individual studies that involved more comprehensive cognitive batteries tended to have small sample sizes [e.g., (19)]. A notable exception is the study by Moore et al. (20), which explored the relationships between performance on a cocktail-party listening task (the digit-triplet test) and a battery of cognitive tests in around 90,000 listeners from the UK Biobank. The authors reported that higher SRTs were associated with worse performance on all cognitive measures, though the raw correlation coefficients were not reported, which makes it difficult to determine the strengths of these associations. Based on our own investigation of the UK Biobank dataset, which revealed that the digit-triplet test had poor test–retest reliability (24), we suspect that the correlations were quite weak. In a metaanalysis of 25 previous studies, Dryden et al. (21) estimated an overall moderate correlation between speech-in-noise performance and cognitive abilities, collapsed across various speech-in-noise tasks, cognitive measures, and listeners with and without hearing impairment. The authors reported correlations with specific cognitive domains. In descending order of strength, these were processing speed, inhibitory control, working memory, episodic memory, and crystallized intelligence. This order does not match our ERV-based order exactly, although in both cases, processing speed appeared to be particularly important.

There is increasing interest in the role of peripheral auditory processing during cocktail-party listening. In particular, cochlear synaptopathy has emerged as a compelling putative mechanism by which the temporal representations of sounds may be disrupted within the peripheral auditory system, degrading cocktail-party listening and leading to real-world hearing problems, without greatly affecting sound sensitivity (13, 16). Crucially, however, there is limited evidence of correlations between putative measures of cochlear synaptopathy and performance on cocktail-party listening tasks or self-reported real-world hearing problems in humans [e.g., (18)]. Measurement insensitivity may be at least partly to blame for these mixed results; that is, non-invasive assays of cochlear synaptopathy may not yet be sensitive enough to yield observable correlations. However, our results suggest an additional possibility, namely that large individual differences in cognitive abilities—which almost always go unmeasured in such studies—may mask these relationships. Future studies seeking to discover relationships between aspects of peripheral auditory function and cocktail-party listening may be better placed to do so if they also measure and adjust for individual differences in listeners' cognitive abilities.

The present study had a few potential limitations. The first was the use of time-reversed maskers. As we discussed previously (24), rendering maskers unintelligible by time-reversing them simplified the task instructions and eliminated some potential sources of confusion, which reduced floor effects and produced SRTs that were better suited to quantitative genetic analysis in this sample. However, one could argue that SRTs measured with time-reversed maskers have less ecological validity than SRTs measured with time-forward maskers because listeners do not encounter time-reversed speech in the real world. This limitation may be important if the masking caused by time-reversed maskers is substantially different in nature to that caused by time-forward maskers, but this does not appear to be true (66). Another potential limitation was that SRTs and HTs were measured using consumer-grade equipment (rather than audiometric equipment) in an ordinary quiet testing room (rather than a sound-attenuated booth). These features make it difficult to compare our listeners' raw SRTs and HTs to those from other psychoacoustic studies, and probably caused them to be higher overall, as well as adding some amount of additional measurement error. However, since the data were transformed prior to analysis, absolute SRT and HT values did not influence our results.

The present study considered the *genetic* factors that jointly influence cocktail-party listening, sound sensitivity, and cognitive abilities, but not the potential *environmental* factors. For example, noise exposure could cause worse SRTs and worse HTs. Unfortunately, we were unable to estimate noise exposure in individual listeners in this study. Previously, we derived an index of neighborhood noise levels based on transportation noise, but this was not associated with either SRTs or HTs (24). Another possible environmental factor that could jointly influence cocktail-party listening, sound sensitivity, and cognitive abilities is cardiovascular health, but we did not observe any correlations with various cardiovascular measures, such as body mass index, in this study. We did find strong effects of sex and age, as expected, and all results reported in the present study controlled for these effects.

In conclusion, the present study revealed that the genetic influences on cocktail-party listening overlap considerably with those on cognitive abilities, including abilities that are not primarily auditory or verbal in nature. These results may have important implications for future studies exploring the physiological and psychological factors that influence real-world hearing problems, as well as their genetic and/or environmental etiologies.

## Data availability statement

The raw data supporting the conclusions of this article will be made available by the authors, without undue reservation.

## Ethics statement

The studies involving human participants were reviewed and approved by the University of Texas Health Science Center at San Antonio and Boston Children's Hospital. The patients/participants provided their written informed consent to participate in this study.

## Author contributions

Study concept and design: SM, DG, and JB. Acquisition, analysis, or interpretation of data: SM, EK, AR, MW, AH, AG, PF, RO, JP, SK, and RD. Drafting of the manuscript and statistical analysis: SM. Critical revision of the manuscript for important intellectual content: SM, EK, JM, AR, MW, AH, AG, PF, RO, JP, SK, HG, RD, JC, JB, and DG. Obtained funding and study supervision: DG and JB. Administrative, technical, or material support: SM, MW, AH, DG, and JB. All authors contributed to the article and approved the submitted version.
